# Circulating miRNome of *Trachemysscripta* after elective gonadectomy under general anesthesia

**DOI:** 10.1038/s41598-021-94113-8

**Published:** 2021-07-19

**Authors:** Edoardo Bardi, Stefano Brizzola, Giuliano Ravasio, Stefano Romussi, Paola Dall’Ara, Valentina Zamarian, Maddalena Arigoni, Raffaele Adolfo Calogero, Cristina Lecchi

**Affiliations:** 1 Dipartimento di Medicina Veterinaria, Università degli Studi di Milano, 20133 Milan, Italy; 2 Department of Biotechnology and Health Sciences, Molecular Biotechnology Center, Università di Torino, 10126 Turin, Italy

**Keywords:** Molecular biology, Non-coding RNAs, Biomarkers

## Abstract

Post-surgical management is an important issue in veterinary medicine, requiring biomarkers with high sensitivity and specificity for timely and effective treatment. Emerging evidence suggests that miRNAs are promising stress- and pain-related markers. The aims were to profile the circulating miRNA signature in plasma of turtles (*Trachemys* *scripta*) and point out potential candidate biomarkers to assess the status of the animal. The plasma of female turtles underwent surgical gonadectomy were collected 24 h pre-surgery, and 2.5 h and 36 h post-surgery. The expression of miRNAs was profiled by Next Generation Sequencing and the dysregulated miRNAs were validated using RT-qPCR. The diagnostic value of miRNAs was calculated by ROC curves. The results showed that 14 miRNAs were differentially expressed over time. RT-qPCR validation highlighted that 2-miR-499-3p and miR-203-5p-out of 8 miRNAs tested were effectively modulated. The Area Under the Curve (AUC) of miR-203-5p was fair (AUC 0.7934) in discriminating pre- and 36 h post-surgery samples and poor for other time points; the AUC of miR-499-3p was excellent (AUC 0.944) in discriminating pre-surgery and 2.5 h post-surgery samples, and fair in discriminating pre-surgery and 36 h post-surgery (AUC 0.7292) and 2.5 h and 36 h post-surgery (AUC 0.7569) samples. In conclusion, we demonstrated for the first time that miRNAs profile changes in plasma of turtles underwent surgical oophorectomy and identified miR-203-5p and miR-499-3p as potential candidate biomarkers to assess animals' status. Further studies are necessary to confirm their diagnostic value and to investigate functional and mechanistic networks to improve our understanding of the biological processes.

## Introduction

MiRNAs are short, non-coding RNA, widely conserved across species, exerting often similar functions in the different animal lineages^1,2^. MiRNAs are epigenetic modulators able to interfere with gene expression by binding with full or partial complementarity to targeted mRNA, leading to either its inhibition of translation or degradation^1^, without inducing any change to the genome. Painful events promote several molecular swings, including the modulation of miRNA expression, which in turn can silence the expression of pro‐ or anti-nociceptive genes; the ability of miRNAs to affect neuronal plasticity and thus to play a pivotal role in pain is reported in several animal models of inflammatory and neuropathic pains and is associated with hyperalgesia and allodynia^3,4^. Pain management is a highly important issue in veterinary medicine, not only for ethical reasons related to animal welfare but also to ensure proper clinical procedures. Nevertheless, animals exhibit complex behavioral and physiological responses to pain, which are ameliorated by analgesics^5^. Pain assessment is challenging in animals and, although several guides based on physical and biological alterations in dogs and cats^6^, horses^7^, and pig^8^ have been developed, the ability of animals to experience pain remains controversial^9^. Pain recognition and treatment in chelonians brings additional challenges: although reptiles have been proved to possess all anatomical and physiological structures necessary to perceive and elaborate nociceptive pain, and to display painful behaviours^10–13^, pain diagnosis and assessment of the efficacy of most analgesic drugs is still rudimentary^10,13–15^. Behavioral changes include reduced mobility and depression, reduced or increased aggression, reduced food intake, increased respiratory rate, unwillingness to swim in aquatic animals. Such changes are not specifically pain-related and can be altered by a wide range of conditions depending on the species, environmental conditions, concurrent diseases, pathophysiological status, and administered therapies^10,12,13^. For these reasons, and unlike in mammalian practice, behavioral ethograms are not completely reliable and are seldom used to assess the status of reptiles in clinical practice, so that clinicians most often provide analgesia extrapolating from other species both the anticipated level of stress and pain and the expected efficacy of analgesic drugs^12^. Thus, identifying objective parameters to quantify the level of pain in turtles is an important step on the path to better understanding and properly treating these animals.

## Materials and methods

### Ethical statement

All applicable international, national, and/or institutional guidelines for the care and use of animals were followed. Samples were collected during pre-surgical clinical evaluation, with written informed consent from the owners. The protocol for care, handling, and sampling of animals defined in the present study was reviewed and approved by the Università degli Studi di Milano Animal Care and Use Committee (protocol no. 107/18). The authors complied with the ARRIVE guidelines (https://arriveguidelines.org/).

### Subjects and sample collection

Fourteen clinically healthy sexually mature female turtles (*Trachemys* *scripta*, age between 10 and 35 years, mean body weight 1423 ± 423 g) were presented for elective surgical gonadectomy for population control in June, July and August 2018 and 2019. Consequently, no animals underwent surgery or were directly used to record data for this study. All the patients were hospitalized one week before surgery and individually housed outdoor; food was withheld 48 h before surgery^16^. Anesthesia was induced by intramuscular (IM) administration of dexmedetomidine (Dexdomitor, Vetoquinol Italia S.r.l., Bertinoro, Italy) 100 µg/kg, ketamine (Nimatek, Dechra Pharmaceuticals PLC, Bladel, Netherland) 3 mg/kg, midazolam (Midazolam IBI, Giovanni Lorenzini S.p.A, Aprilia, Italy) 0.5 mg/kg and alfaxalone (Alfaxan, Jurox (UK) Limited, Malvern, UK) 8.5 mg/kg; animals were intubated with a 1.5–2.0 mm Cole’s endotracheal tube and intermittent positive-pressure ventilation was manually provided (2 breaths per minute) administering 100% O_2_ without inhalant anesthetics; such protocol was selected as part of a separate study. Monitored physiological and clinical parameters during general anesthesia were palpebral and corneal reflexes, limb withdrawal latency, cloacal temperature, and heart rate via ECG^13^. Oophorectomy was achieved by endoscope-assisted prefemoral coeliotomy as previously described^17^. Dexmedetomidine and midazolam were reversed respectively with atipamezole (Antisedan, Vetoquinol Italia S.r.l., Bertinoro, Italy) 1 mg/kg and flumazenil (Flumazenil, B. Braun Melsungen AG, Melsungen, Germany) 0.05 mg/kg administered half intravenously in the cervical sinus and half IM in the pectoral muscle 20 min after the end of the procedure. After surgery, every animal was kept hospitalized for one week in order to monitor for post-surgical complications.

Blood samples were collected at three time points (24 h pre- and 2.5 h and 36 h post-surgery) from the cervical sinus into Monovette EDTA tubes (Sarstedt Company, Nümbrecht, Germany) and centrifuged at 800 × *g* for 15 min. Plasma was stored at − 80 °C until RNA extraction. Pre-operative hematological analyses were performed for each animal as previously suggested^18,19^. The sequencing analysis was performed on the plasma of 8 patients at three time points, while the validation step by qPCR was performed on the 8 sequenced samples and on a separate independent set of 6 samples at three time points.

### miRNA extraction using two commercial kits

To identify the best kit to isolate microRNAs from turtle plasma, a comparison of two commercial kits for microRNA extraction was performed. Small RNAs were extracted using miRNeasy Serum/Plasma kit (Qiagen, Cat. No 217184) and microRNA Concentrator kit (A&A Biotechnology, Cat. No 035-25). Only plasma collected at 2.5 h post-surgery has been included in this analysis. Plasma was thawed on ice and centrifuges at 3000 × *g* for 5 min at 4 °C. An aliquot of 100 µL per sample was transferred in a new tube and smallRNA was extracted using the two kits, in accordance with the manufacturer’s instruction. The *Caenorhabditis* *elegans* miRNA cel-miR-39 (25 fmol final concentration) (Qiagen, Cat. No 219610) was used as synthetic spike-in control. To compare the performance of the kits, the spike-in cel-miR-39 was quantified by RT-qPCR as explained below, and the Cq values were compared. After NGS, the analysis was carried out on the same samples targeting DE-miRNAs, to confirm the results.

### Next generation sequencing (NGS)

The RNA quality and quantity were verified according to MIQE guidelines^20^. The RNA concentration was quantified by Qubit^®^ 2.0 Fluorometer with Qubit^®^ microRNA Assay Kit (Invitrogen, Cat. No. Q32880). An NGS was performed on the plasma of 8 patients at three time points. Small RNA transcripts were converted into barcoded cDNA libraries. Library preparation was performed as previously reported^21^ using the NEBNext Multiplex small RNA Library Prep Set (Cat No NEB#E7560) for Illumina and run on the NextSeq500 (Illumina Inc., USA).

### Computational analysis

The output of NextSeq500 Illumina sequencer was demultiplexed using bcl2fastq Illumina software embedded in docker4seq package^22^ (https://github.com/kendomaniac/docker4seq). miRNA expression quantification was performed using the workflow previously described^21^ (https://github.com/kendomaniac/docker4seq). In brief, fastq files were quality checked (QC) using FastQC software (http://www.bioinformatics.babraham.ac.uk/projects/fastqc/). Reads shorter than 14 nt were discarded. The QC-passed reads were clipped from the adapter sequences using Cutadapt^23^, by imposing a maximum error rate in terms of mismatches, insertions, and deletions equal to 0.15. Since *Trachemys* *scripta* miRNA sequences are not available in a public repository, sequences were mapped to *all* *saurians* (aca, cpi, ami, oha) precursors miRNAs available in miRBase 22.0 (http://www.mirbase.org/) based on phylogeny^24^. The alignment was performed using BWA^25^ algorithm v. 0.7.12 with the default settings. Annotation and quantification of miRNAs were done as described^26^. The detected counts were organized in a table including all analysed samples. For visualization purposes, only CPM (counts per million reads) were used. Differential expression analysis was evaluated using DESeq2 Bioconductor package^27^ implemented in docker4seq package (https://github.com/kendomaniac/docker4seq). Differential expression analysis was done using the above-mentioned counts' table comparing different time points using as threshold an adjusted *P-*value ≤ 0.1 and an absolute log2 Fold Change (log2FC) ≥ 1. In case a miRNA was detected in *Chrysemys* *picta* (cpi) and in other saurian only the *Chrysemys* *picta* was reported.

### miRNA quantification by qPCR

Reverse transcription was performed using TaqMan Advanced miRNA cDNA Synthesis Kit (Cat. No A28007, Applied Biosystems), following the manufacturer’s instruction.

To validate NGS results, qPCR was performed following the MIQE guidelines^20^. Target DE-miRNAs, included miR-187-5p, miR-143-5p, miR-92b-5p, miR-218-2-3p, miR-203-5p, miR-138-1-3p, miR-196-2-3p, miR-499-3p, were custom designed by ThermoFisher Scientific service. The assay ID of cel-miR-39 probe was 478293_mir.

The quantitative reaction was performed in a scale down-reaction volume (15 µl) on CFX Connect Real-Time PCR Detection System (Biorad) using 7.5 µl of 2X TaqMan Fast Advanced Master Mix (Cat. No 4444557), 0.75 µl of miRNA specific TaqMan Advance assay (20×), 1 µl of cDNA and water to reach the volume. The thermal profile was 50 °C for 2 min, 95 °C for 3 min and 40 cycles of 95 °C for 15 s and 60 °C for 40 s. No-RT controls and no- template controls were performed. Data were normalized relative to the cel-miR-39 expression on Bio-Rad CFX Maestro™ Software 1.0 using the 2^−ΔΔCq^ method.

### Statistical analysis

Statistical analysis was performed using XLStat software for Windows (2014; Addinsoft, New York, USA).

Data were tested for normality using the Shapiro–Wilk test; as the data were not normally distributed, the nonparametric Wilcoxon signed-rank test for paired samples was applied. Statistical significance was accepted at *P*-value ≤ 0.05. Receiver Operating Characteristic (ROC) analysis was performed to determine the diagnostic accuracy of target that statistically differed between groups. The diagnostic value was calculated for that miRNA that showed a significant differential expression in the blood of turtles, namely miR-499-3p and miR-203-5p. The ROC analysis was carried out by plotting the true positive (sensitivity) versus the false positive (1-specificity).

## Results

Physiological and clinical parameters remained stable throughout the procedure for all patients. All the animals returned to normal levels of activity and spontaneous feeding within 12 h after surgery, and no post-surgical complication occurred during the 1-week follow-up period.

### A&A Biotechnology kit was selected to extract miRNAs from turtle plasma

To assess the success of miRNA extraction using the two kits, cel-miR-39 was quantified by RT-qPCR on samples collected at 2.5 h post-surgery, as a preliminary step before NGS. At the end of the study, the good performance of the selected kit was confirmed comparing the Cq values of DE-miRNAs at 2.5 h post-surgery starting from the same extracts. Overall, the kit by A&A Biotechnology was the best with the lower Cq values across the 5 miRNAs (*P* < 0.031) (Supplementary Fig. 1). In detail, the differences of Cq values between Qiagen and A&A Biotechnology kits were 7.33, 6.72, 4.36, 8.66 and 2.84 for cel-miR-39, miR-499-3p, miR-203-5p, miR-92b-5p and miR-138-1-3p, respectively. Based on the results on cel-miR-39 the kit by A&A Biotechnology was selected.

### Determination of miRNAs profile and identification of DE-miRNAs

**Figure 1:**
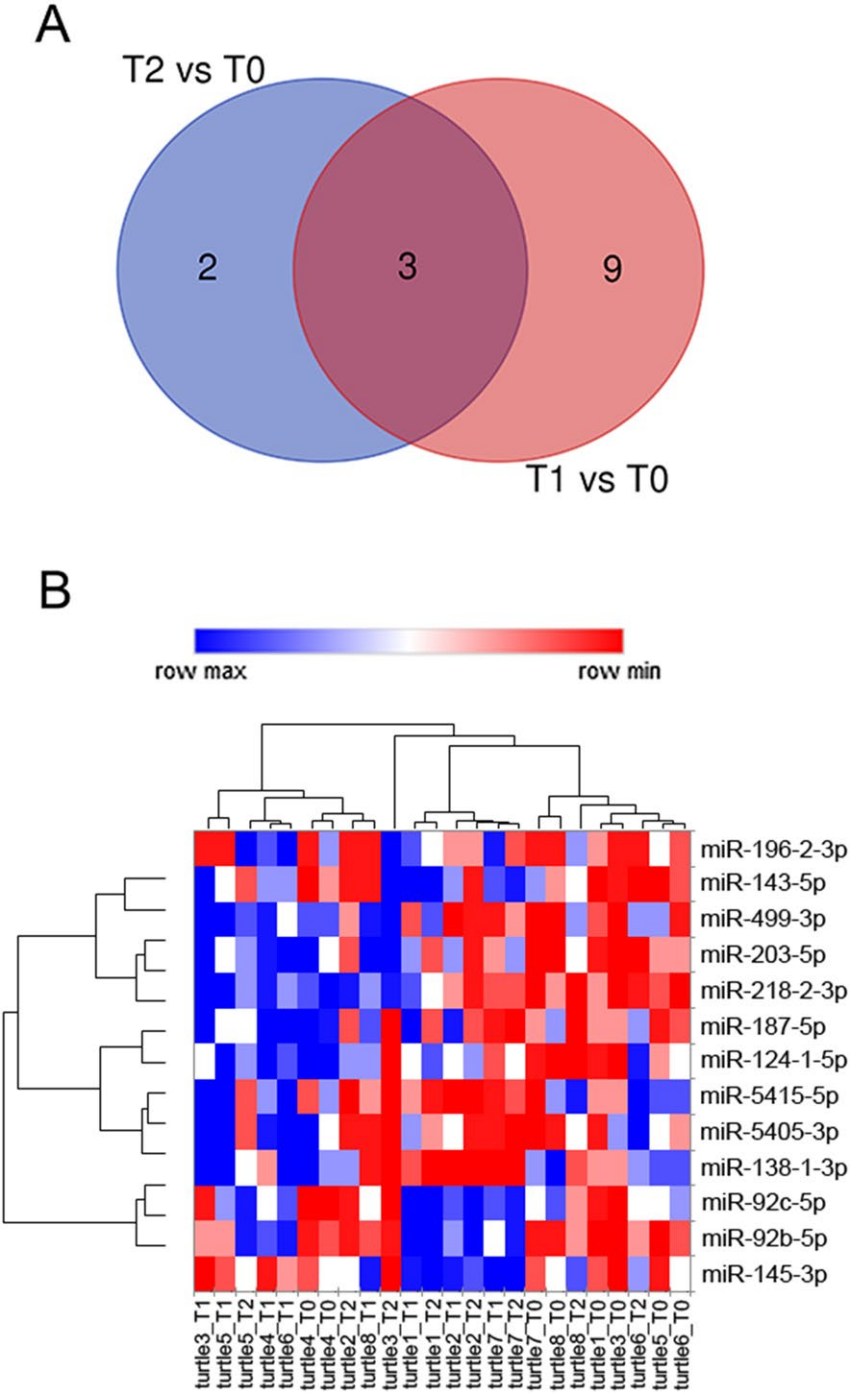
Identification of DE-miRNAs on plasma of turtle comparing pre-surgery (T0) and 2.5 h (T1) and 36 h (T2) post-surgery. (**A**) Venn diagram showing DE- miRNAs; (**B**) heat-map DE-miRNAs.

To characterize miRNA expression profiles of turtle plasma, a small RNA-seq was carried out on RNA extracted from twenty-four (eight patients at three time points) samples. After RNA extraction, small RNAs were size-selected on gel and sequenced on the NextSeq500 sequencer (Illumina). Primary data are deposited on GEO database (GSE175466). A number of reads per sample, varying from 182,606 to 26,665,830, was obtained (Supplementary Table 1). Counts table was used to detect differentially expressed (DE)-miRNAs via DESeq2 analysis^27^. Furthermore, the differential expression analysis with DESeq2, performed on 3988 saura (*Anolis carolinensis, Chrysemys picta, Alligator mississippiensis, Ophiophagus hannah*) miRNAs, detected 14 differentially expressed miRNAs (|log_2_FC|≥ 1, FDR ≤ 0.1). Thirteen DE-miRNAs were up-regulated after surgery, while only one, miR-138-1-3p, was down-regulated. Nine out of fourteen were found differentially expressed between 2.5 h post-surgery and pre-surgery. Two out of fourteen were detected as differentially expressed between 36 h post-surgery and per-surgery. Three miRNAs were shared between 2.5 h post-surgery and 36 h post-surgery (Table 1; Fig. 1A,B).

**Table 1:** Differentially expressed miRNAs. T0 = 24 h pre-surgery; T1 = 2.5 h post-surgery; T2 = 36 h post-surgery.

	Log_2_Fold Change	*P* value
**De-miRNAs T0 vs T1**		
mir-125b-1-5p	1.01	0.01918
mir-92c-5p	1.02	0.03052
mir-5415-5p	1.07	0.02528
mir-187-5p	1.12	0.00538
mir-5405-3p	1.15	0.01288
mir-143-5p	1.15	0.00082
mir-218-2-3p	1.24	0.00839
mir-29a-1-3p	1.27	0.01450
mir-203-5p	1.37	0.00232
**De-miRNAs T0 vs T2**		
mir-138-1-3p	-1.29	0.00149
mir-196–2-3p	1.04	0.01555
**De-miRNAs T1 and T2 vs T0**		
mir-145-3p	1.14	0.00194
mir-92b-5p	1.44	0.00036
mir-499-3p	1.36	0.00176

### Validation of DE-miRNAs: miR-203-5p and miR-499-3p are modulated after surgery

**Figure 2:**
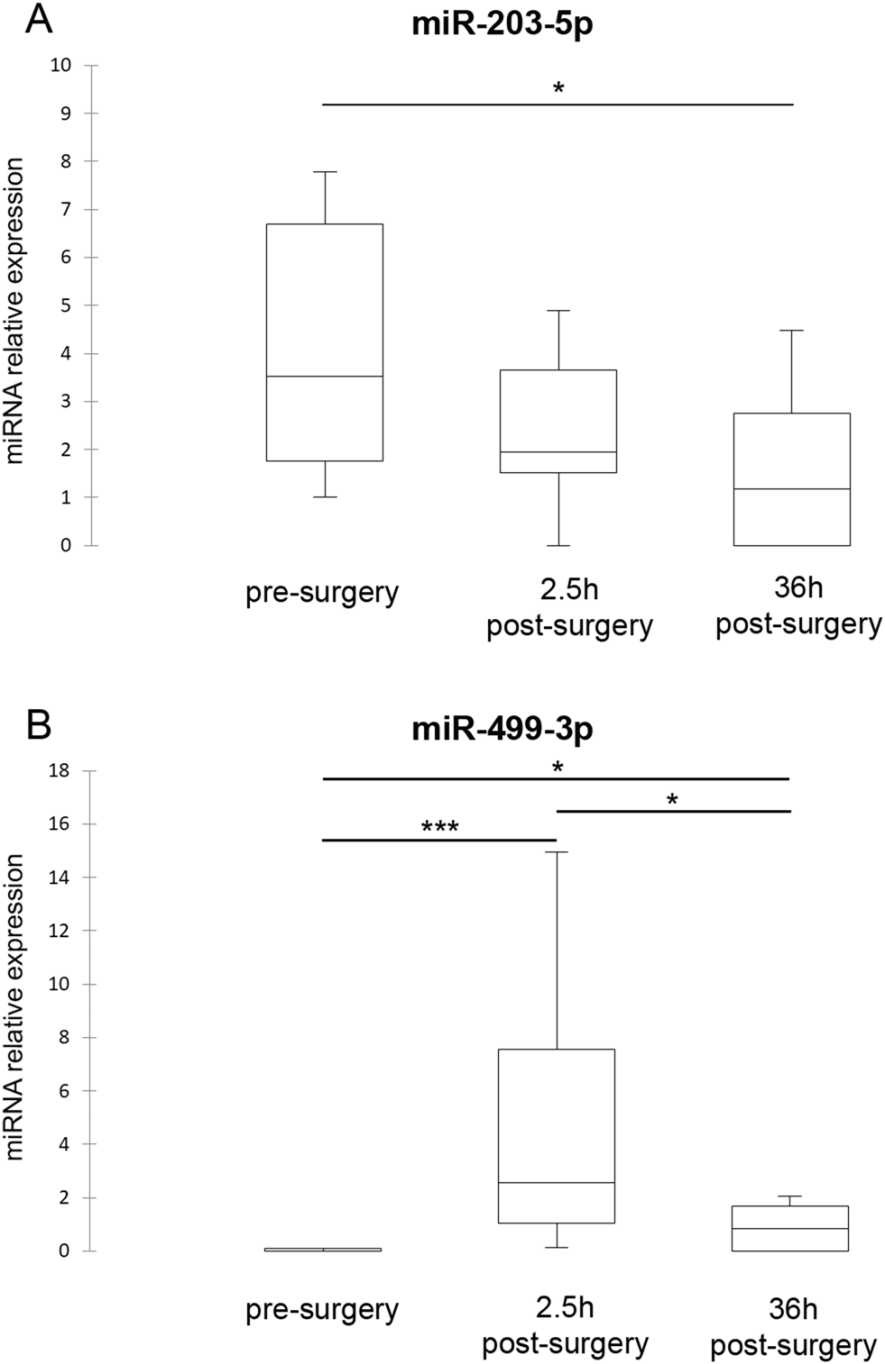
Box plots of DE-miRNAs pre- and 2.5 h and 36 h post-surgery. Significance was declared at *P* < 0.05 (*), *P* < 0.01 (**) and *P* < 0.001 (***). Black lines inside the boxes mark the medians.

RT-qPCR validation was performed on the 8 sequenced samples and on a separate independent set of 6 samples at three time points. To validate the sequencing results, eight differentially expressed (DE)-miRNAs were selected. MiRNA levels were normalized to that of cel-miR-39, an artificial spike-in that was used as an internal control. Four (miR-92b-5p, miR-138-1-3p, miR-203-5p, and miR-499-3p) out of 8 selected miRNA targets were detected in plasma samples, among which only miR-203-5p and miR-499-3p were significantly differentially expressed over time. In details, miR-203-5p was down-expressed at 36 h post-surgery compared with pre-surgery (ratio_pre/36 h post_ = 4.3; *P* = 0.021); miR-499-3p was higher at 2.5 h post-surgery compared to pre-surgery (ratio_2.5post/pre_ = 21.9; *P* = 0.0007) and 36 h post-surgery (ratio_2.5 h post/36 h post_ = 2.9; *P* = 0.047), and 36 h post-surgery was overexpressed compared to pre-surgery (ratio_36 h post/pre_ = 7.5; *P* = 0.0007) (Fig. 2). The Cq of the other four miRNAs (miR-143-5p, miR-187-5p, miR-196-2-3p, and miR-218-2-3p) was ≥ 35, thus they were not quantified.

### Diagnostic performance of circulating miR-203-5p and miR-499-3p

**Figure 3:**
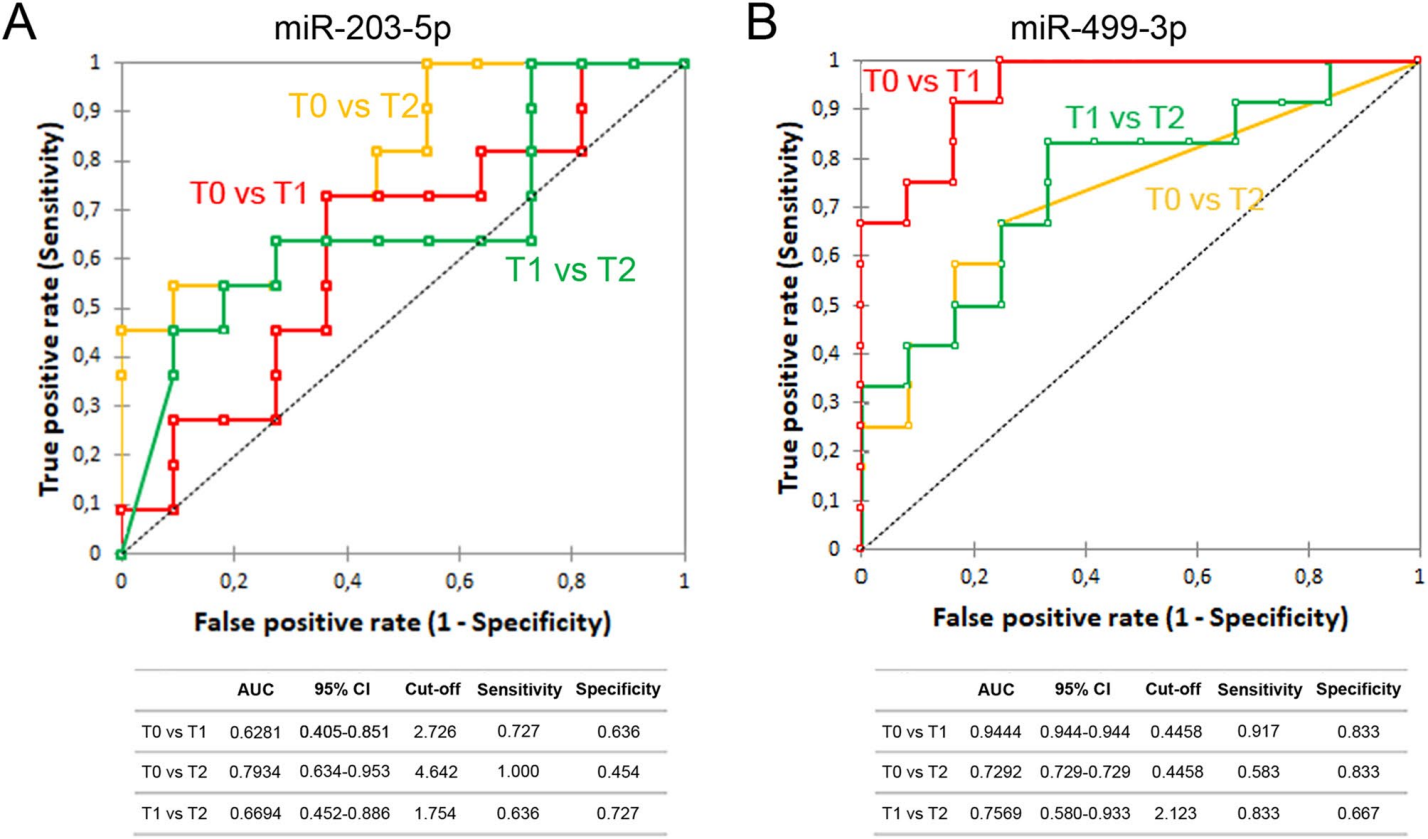
Receiver-operator characteristic (ROC) curve analysis of DE-miRNAs. AUC, area under the curve; CI, confidence interval. T0 = pre-surgery; T1 = 2.5 h post-surgery; T2 = 36 h post-surgery.

Since circulating miR-203-5p and miR-499-3p were differentially expressed over time, further analysis was performed on these miRNAs. To evaluate the diagnostic value, ROC curve analysis was performed and the associated area under the curve (AUC), where an area of 1 represents a perfect test and an area of 0.5 a worthless test, was used to confirm the diagnostic potency. The cut-off point was set to maximize the sum of sensitivity and specificity. The ability of miR-203-5p was fair in discriminating pre-surgery and 36 h post-surgery samples (AUC 0.7934; 95% CI 0.634–0.953) and poor for other time points (Fig. 3A). The ability of miR-499-3p was excellent in discriminating pre-surgery and 2.5 h post-surgery (AUC 0.944; 95% CI 0.944–0.944), and fair in discriminating pre-surgery and 36 h post-surgery (AUC 0.7292; 95% CI 0.729–0.729) and 2.5 h and 36 h post-surgery (AUC 0.7569; 95% CI 0.580–0.729) (Fig. 3B).

## Discussion

Novel biomarkers with high sensitivity and specificity are required for assessing the status of post-surgical animals. Emerging evidence suggests that miRNAs are promising stress- and pain-related markers with the advantage of high sensitivity and specificity in different animal species^28,29^. However, no investigations have been conducted on circulating miRNAs associated with post-surgical chelonians so far.

In the present study, prefemoral gonadectomy of female pond sliders (*Trachemys* *scripta*) has been selected as a surgical model, based on the hypothesis that it may affect plasma molecular markers. A multistep approach, using Next Generation Sequencing as a first step to identifying differentially expressed miRNAs in plasma at three-time points, then validating them by RT-qPCR on the sequenced samples and a separate independent set of samples, was adopted. Our results provided for the first time the evidence that (I) the expression level of circulating miRNAs changes in the plasma of pond sliders underwent surgical castration; (II) the expression level of miR-203-5p decreased 4.3-folds at 36 h post-surgery compared with pre-surgery level, and that of miR-499-3p increased 21.9-folds at 2.5 h post-surgery compared with pre-surgery and decreased to a level similar to that of pre-surgery at 36 h post-surgery; (III) diagnostic accuracy of miR-203-5p was fair in discriminating pre- and 36 h post-surgery animals and that of miR-499-3p was excellent in discriminating animals over time.

The successful purification of miRNAs from small volumes of biofluids, including plasma, is challenging, especially in turtles, for which, to the best of our knowledge, no investigations on circulating miRNAs have been previously performed. As previously reported^30,31^, miRNA recovery can be influenced by the isolation method and a careful selection of the RNA isolation method should be explored to identify the best approach for own samples. We compared two column-based commercially available kits developed for miRNAs extraction from biofluids and then we evaluated the qPCR performance using Cq values. The results showed that the A&A Biotechnology kit allowed us to obtain lower Cq values than the Qiagen kit; thus, we identified A&A Biotechnology kit to isolate miRNAs from the plasma of turtles. Sequencing data supplied a list of differentially abundant miRNAs but the validation step using RT-qPCR showed that the plasma miRNAs rate at three-time points was not statistically significant for 6 out of 8 miRNAs tested. The technical bias inherent in sequencing technologies may provide misshape results. A substantial distortion between miRNA levels in NGS data and true miRNA abundancy may occur using Illumina sequencing technology^32^, thus the validation step by qPCR is always advisable. Another issue concerns the analysis of miRNAs in plasma, which is more challenging than conventional intracellular miRNAs. The miRNAs library preparation from plasma suffers from two main critical issues: (i) lower number of miRNAs compared to the intracellular ones, and (ii) higher background due to the presence of degraded RNA fragments in the blood. Especially the contamination of fragmented unspecific RNA massively reduces the number of reads mappable on miRBase precursors. The protocols for preparing miRNAs’ libraries need high-quality total RNA to obtain high recovery of miRNA reads from intracellular total RNA. Unfortunately, when working with circulating miRNA it becomes impossible to avoid the contamination of fragmented unspecific RNA. To limit the impact of this issue on the number of identified miRNAs, our sequences were mapped to all saurian precursors miRNAs available in miRBase 22.0; nevertheless, the fraction of reads mapping represented only a very small part of the total sequenced reads.

Since few specific diagnostic tests are available and few drugs have been investigated in reptiles, dosages are most of the time empirically extrapolated without proper notions on their effectiveness, which is a particular concern for painkilling drugs, especially as it is harder to understand the welfare status of reptiles^33^. It has been recognized that reptiles are sentient, being able to feel emotion, pleasure, fear, stress, grief, and pain, as recently well summarized by Lambert and colleagues^34^. *Trachemys* *scripta* has been introduced in Europe from North America since the mid-‘80s. It has competitive advantages over native species such as the European pond turtle (*Emys* *orbicularis*), including lower age at maturity, higher fecundity, and larger adult body size^35^. Turtles may compete for food, egg-laying sites, or basking places, the best of which are occupied by *T.* *scripta*^35^, thus this species is included in the list of invasive alien species by the EU regulation 2016/1141. Official eradication programs have not yet been established. Surgical gonadectomy has been proposed as means for population control, but several obstacles limit successful discomfort recognition and treatment, such as subjectivity in pain assessment, and limited knowledge of analgesic efficacy^10,15,36^. Usually, discomfort in non-verbal patients is assessed by semi-objective scales, using both behavioral and physiological parameters believed to be correlated with a stressful or painful condition^10,12^; unfortunately, in reptiles, such parameters aren’t exclusive nor strongly correlated to discomfort^12^, requiring careful and often time-consuming evaluation. Thus, the identification of proper biomarkers to evaluate the chelonians’ status may support the clinical decision-making process. The here selected anesthetic protocol consisted of a combination of a benzodiazepine (midazolam), an α_2_-agonist (dexmedetomidine), a neuroactive steroid (alfaxalone), and a dissociative agent derived from phencyclidine (ketamine). Benzodiazepines and alfaxalone both act on inhibitory neurotransmitter gamma aminobutyric acid complex (GABA) receptors^37,38^: midazolam enhancing central and peripheral GABA_A_ receptors^39^, and alfaxalone acting on central GABA receptors^37^. Alpha-2 agonists are used in combination with other molecules for sedation and myorelaxation^40,41^, stimulating central and peripheral α_2_-adrenergic receptors decreasing excitability, and provide analgesia^42^; their effects can be antagonized with specific antagonists such as atipamezole^40^. Ketamine, acting as *N*-methyl-d-aspartate (NMDA) glutamate central and peripheral receptor antagonist, is a dissociative anesthetic drug frequently used in reptilian practice in combination with other drugs (multimodal anesthesia regimen) to reduce side effects and recovery time^13,40,43–45^. Ovariectomy in animals is a relatively standardized source of moderate soft tissue pain, making it suitable for clinical studies^46^. In the present study, for ethical reasons, it was decided to provide analgesic drugs, and since GABA-agonists do not display effective analgesic property, selected molecules for this purpose was dexmedetomidine and ketamine. Analgesic efficacy of ketamine and α_2_-agonist has been well demonstrated in mammalian practice^47^; in reptiles, such properties are still controversial, but indications are pointing to the potential use of α_2_-agonist as analgesic supplement^11–13,42,48–50^. The increase in miR-499-3p expression at T1 can be thus explained by the depletion of the activity of dexmedetomidine after reversal with atipamezole, while the decrease 36 h after surgery is likely correlated to the normal decrease of acute pain caused by the procedure. The absence/low level of discomfort at this time point could be confirmed by the fact that, even if the behavioral ethogram proposed by Kinney and co-workers^10^ was not applied, all the animals showed normal behavior and feeding 24 h after the surgery. The miR-203-5p can be considered as a "negative biomarker". Indeed, it is found in a considerable amount at pre-surgery, lowers at 2.5 h post-surgery, and becomes significantly lower (*P* = 0.021) at 36 h post-surgery compared to the pre-surgery level. Considering that no NSAIDs were administered in the post-operative period in this study, this negative biomarker could be influenced by surgery, inflammation, drugs, pain and, more in general, by stress and discomfort. Focusing on surgical inflammation, it usually refers to the initial physiological response to tissue damage. Acute inflammation begins within seconds to minutes following an injury to tissues and increases in the following hours^51^. The miR-203-5p production, high in normal conditions, may be suppressed by several factors, including the presence of pro-inflammatory molecules in post-surgical course. Further studies are necessary to explore and confirm this hypothesis, for example by the administration of NSAIDs to turtles in the post-operative period.

## Conclusions

To support the clinicians in the management of post-surgical chelonians, we explored the potential use of miRNAs as markers to assess and monitor the post-surgical status of turtles (*T.* *scripta*), demonstrating that miR-499-3p and miR-203-5p might be further explored as promising candidate biomarkers. MiR-499-3p and miR-203-5p are modulated after surgery, representing potential candidate biomarkers of chelonians’ discomfort. Nevertheless, the pathophysiological role played by miR-499-3p and miR-203-5p must be yet fully elucidated. Although this study has provided new and important insights, further experiments are required to identify and validate the specific elements these miRNAs are useful for measuring. Since the nucleotide sequence of miR-499-3p and miR-203-5p are not conserved between the turtle and human and other species for which potential target genes and functional information are available on public databases, Gene Ontology could not be carried out.

## Supplementary Information

Supplementary Information.

Supplementary Figure.
