# Supplementary material for: Circulating miRNome of Trachemysscripta after elective gonadectomy under general anesthesia
Source: Sci Rep. 2021 Jul 19;11:14712. doi: 10.1038/s41598-021-94113-8 (PMC8289937; doi:10.1038/s41598-021-94113-8)
Supplement: Supplementary file 2 — Supplementary Figure. [file 41598_2021_94113_MOESM2_ESM.pdf]

Supplementary figure 1.

Circulating miRNome of *Trachemys scripta* after elective gonadectomy under general anesthesia

Edoardo Bardi[1], Stefano Brizzola[1], Giuliano Ravasio[1], Stefano Romussi[1], Paola Dall'Ara[1], Valentina Zamarian[1], Maddalena Arigoni[2], Raffaele Adolfo Calogero[2], Cristina Lecchi[1*].

[1]Dipartimento di Medicina Veterinaria, Università degli Studi di Milano, Milano, 20133, Italy

[2]Molecular Biotechnology Center, Department of Biotechnology and Health Sciences, Università di Torino, 10126, Italy

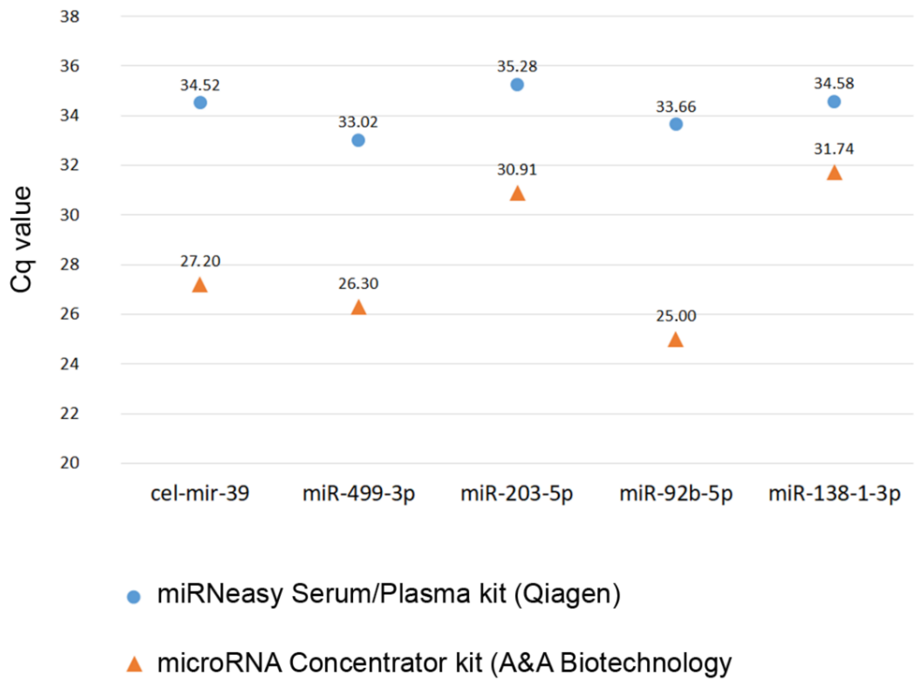

Supplementary figure 1. Evaluation of extraction kit performance. Cq values of DE-miRNAs at 2.5 h post-surgery quantified by RT-qPCR.
